# Chaotic dynamics underpins the slow oscillation of general anesthesia and nonREM sleep

**DOI:** 10.1186/1471-2202-13-S1-F3

**Published:** 2012-07-16

**Authors:** Moira L Steyn-Ross, D Alistair Steyn-Ross, Jamie W Sleigh

**Affiliations:** 1 School of Engineering, University of Waikato, Hamilton 3240, New Zealand; 2 Waikato Clinical School, University of Auckland, Hamilton 3240, New Zealand

## 

Electrical recordings of brain activity show that entry into anesthetic unconsciousness is signposted by the emergence of large, slow oscillations of electrical activity (~1 Hz) that appear very similar to the slow waves observed in natural sleep. In this phase, populations of cortical neurons periodically switch between hyperpolarized inactivity (“down” state), and wake-like depolarized activation (“up” state) [1]. The origin of the slow oscillation has not yet been unambiguously established, and remains an area of intense research and debate [2,3]. Here we suggest a novel mechanism in which the up- and down-states are generated spontaneously by emergent chaotic waves of spatiotemporal activity that sweep the cortex. We present a mean-field model of the cortex in which populations of neurons are densely interlinked by both chemical synapses—including idealized long-range spatially heterogeneous connections—and by direct electrical connections forming a continuous network of interneuronal gap junctions. Anesthetic effect is modeled as a moderate reduction in inhibitory diffusion, paired with an increase in inhibitory postsynaptic potential. We explore model dynamics in the vicinity of a general-anesthetic induced transition from wake to coma. In this region the system is poised at a codimension-2 point where competing Turing (spatial) and Hopf (temporal) instabilities co-exist. We argue that normal functioning of the resting “default-wake” brain requires a delicate balance between these instabilities. Reduction of gap-junction diffusivity disturbs the balance in favor of the Hopf instability, eventually predicting global seizure in the limit of severe imbalance.

Our cortical model predicts that introduction of anesthetic to the awake brain will force a subtle rebalancing of dynamic pressures resulting in a coma state that is characterized by emergent low-frequency oscillations whose dynamics is chaotic in time and space: see Fig. 1. We quantify cortical dynamics in terms of a phase coherence measure and demonstrate that the model-predicted turbulent slow-wave state is characterized by low phase coherence. This prediction is supported by clinical studies of phase synchronization changes in EEG during induction of propofol anesthesia [4].

**Figure 1:**
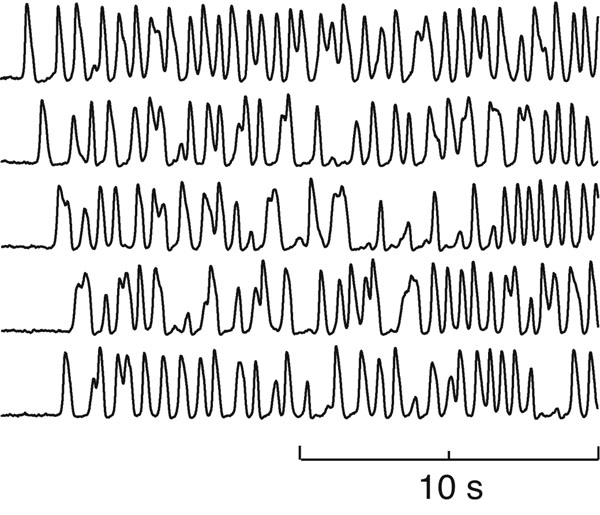
Spontaneous slow-wave oscillations in cortical firing-rate patterns during 20 s of simulated anesthesia. Traces were recorded from five equally-spaced points lying along the midline of the 25-×25-cm simulated cortical grid. Time-series are chaotic in space and time.

## Conclusion

A spontaneous, spatiotemporally chaotic state—generated by nonlinear Turing–Hopf interaction—is the underlying mechanism for the slow oscillation observed in general anesthesia. A similar transition to low-frequency chaos may also occur in natural nonREM sleep, and if so, may have significant implications for synaptic downscaling and memory processing hypothesized to occur during deep sleep.
